# U-DAVIS-Deep Learning Based Arm Venous Image Segmentation Technique for Venipuncture

**DOI:** 10.1155/2022/4559219

**Published:** 2022-10-04

**Authors:** Avik Kuthiala, Naman Tuli, Harpreet Singh, Omer F. Boyraz, Neeru Jindal, Ravimohan Mavuduru, Smita Pattanaik, Prashant Singh Rana

**Affiliations:** ^1^Thapar Institute of Engineering and Technology, Patiala 147001, India; ^2^Research and Development Centre, Gebze, Kocaeli 41400, Turkey; ^3^Postgraduate Institute of Medical Education and Research, Chandigarh 160012, India

## Abstract

Arm Venous Segmentation plays a crucial role in smart venipuncture. The difficulties faced in locating veins for intravenous procedures can be diminished using computer vision for vein imaging. To facilitate this, a high-resolution dataset consisting of arm images was curated and has been presented in this study. Leveraging the ability of Near Infrared Imaging to easily detect veins, ambient lighting conditions were created inside a small enclosure to capture the images. The acquired images were annotated to create the corresponding masks for the dataset. To extend the scope and assert the usability of the dataset, the images, and corresponding masks were used to train an image segmentation model. In addition to using basic preprocessing and image augmentation based techniques, a U-Net based algorithmic architecture has been used to facilitate the task of segmentation. Subsequently, the results of performing image segmentation after applying the preprocessing methods have been compared using various evaluation metrics and have been visualised in the study. Furthermore, the possible applications of the presented dataset have been investigated in the study.

## 1. Introduction

Venipuncture, the process of puncturing the vein either to extract blood or to perform intravenous therapy, is one of the most routinely performed procedures in healthcare. This procedure is most commonly performed on the antecubital fossa region which houses 3 important veins: median cubital vein, cephalic vein, and the basilic vein. The challenges faced by medical personnel to visualise and locate veins in the arm lead to the development of computer aided systems to assist in locating veins with ease. However, the scope of leveraging computer vision techniques for vein imaging in the arm region is restricted by the limited availability of relevant image datasets. Therefore in this study, a new technique for arm vein imaging is proposed and a primary dataset hence produced is presented to minimize the difficulties of vein imaging. In this way, it is aimed to find the veins more easily by the healthcare personnel by contributing to the vein imaging studies. The article is structured as follows: Section 1.1 provides a review of the existing work carried out on human vein imaging (hand and finger veins), Section 2 describes the vein image acquisition system; Section 3 discusses image preprocessing techniques; Section 4 describes the experimental validation procedures; the results are discussed in Section 5, followed by Section 6 which concludes the article.

### 1.1. Existing Work

Many imaging techniques have been proposed to aid in locating the veins [1]. One of these techniques is infrared vein imaging. Infrared rays with wavelengths of 740 nm and 1100 nm are used effectively in vein imaging systems. Subcutaneous vein patterns become visible with infrared illumination [2]. Rays with near infrared wavelengths in the range of 700 nm–850 nm can penetrate the skin up to a depth of 5 mm [3]. Veins carrying oxygen-free blood absorb near infrared rays, while other skin and peripheral tissues reflect these rays. Because of these properties, blood vessels appear darker when captured with an infrared Camera [4]. For these reasons, the near infrared imaging technique has been used in this study.

Although near infrared illumination is a low cost and useful solution, the low resolution of the images is one of its negative aspects. There are many digital filters available to overcome this problem. Various preprocessing techniques such as Adaptive histogram equalization (AHE) [5] and Contrast limited adaptive histogram equalization (CLAHE) [6] are known to improve the image quality. In this study, both these methods were tested on arm vein images and performance analysis have been made.

Yıldız and Boyraz [7] designed a raspberry pi based low-cost vein imaging device in their study. The overall cost of the system is around $ 75. The images captured from the infrared camera were subjected to grey level transformation, CLAHE, median filter, adaptive thresholding, and various morphological processes, respectively. All image processing techniques are implemented using open source OpenCV and *Python* language. In this way, it is aimed to segment the vascular patterns clearly. As a result, the segmented images were checked by the specialist and their success rates were analysed.

Mela et al. Combined visible spectrum (VIS) and near-infrared (NIR) methods in order to make vascular imaging more prominent and to increase diagnostic capacity. In studies performed on the images of the vessels collected over 25 subjects, it was observed that the vascular visibility increased 2 times compared to the naked eye. The accuracy, functionality, and ease of use of the developed device have made it useful for vascular imaging and biometric analysis [8].

Ayoub et al. used a high-resolution infrared camera, vein warmer, and image contrast enhancer (CLAHE) system to improve the visual appearance of vein imaging. It was concluded that the temperature increases positively affected the vein imaging [9]. The methodology and the effectiveness of this enhancement effect in vein detection have been thoroughly discussed in Sections 3 and 5, respectively.

Kim et al. have developed a new low cost vein imaging device. In the study, they investigated from which angle the infrared illumination penetrates the skin better. It has been aimed to visualise even the vascular areas in the arm areas with excess fatty tissue [10].

Ton and Veldhuis have curated a vascular pattern image dataset of fingers using a customized image capturing device which uses eight 850 nm LEDs for illumination. Furthermore, the camera is fitted with an infrared filter to eliminate interfering visible light beyond the wavelength of 930 mm. Thus, enabling them to capture vascular patterns of fingers which are mostly invisible to the naked eye [11]. However, the design of this device allows for only one entry point, making it impractical for use on the human arm.

Dhakshayani and Yacin have developed a portable, cost-effective and reliable vein imaging device to overcome the difficulties in finding veins regardless of age and tissue thickness. LEDs with wavelengths of 740, 765, 770, and 780 nm were used in the infrared illumination system. In this way, it is aimed to better visualise the vascular areas that are difficult to see [12].

Ronneberger et al. introduced the U-Net, which used skip connections to achieve a higher localisation accuracy than a pre-existing sliding window based Convolutional Neural Network. They showed that the U-Net architecture produced precise segmentations even with a low amount of training samples, especially for biomedical image applications [13]. Du et al. have presented a detailed review discussing the importance of U-Net architecture in medical image segmentation. It was shown that U-Net based methods have been successful in producing accurate masks for the segmentation of various tumors, sections of the heart, liver, etc., even with a small amount of available training data [14]. Therefore in this study, a U-Net based architecture has been used to carry out image segmentation on the collected vein images for demonstrating the usability of the introduced dataset.

## 2. Data Acquisition

### 2.1. Description of the Dataset

The presented dataset contains 1850 forearm vein images having a resolution of 2592 × 1944 pixels (5 MP), stored in JPG format.

**Figure 1:**
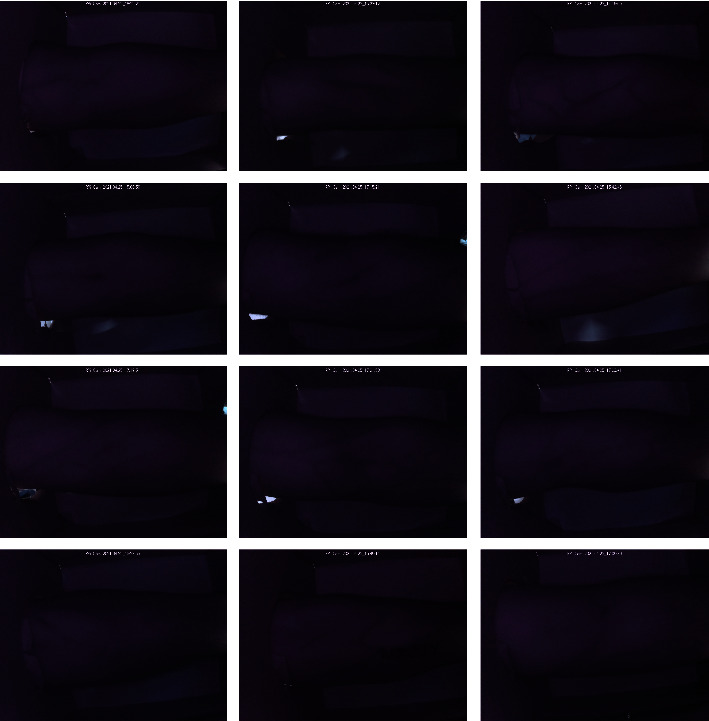
Image samples from the collected dataset.

The entire data collection process was carried out over a span of 2 weeks in the Northern Railway Health Unit, Ludhiana, India. 450 individuals aged 25–75 participated in the procedure. The representation of age groups in the dataset is roughly normally distributed, however the ratio of male to female representation in the dataset is 4 : 1. All participants were informed about the noninvasive and nonharmful nature of the data collection procedure and gave written consent agreeing to volunteer for the same.  Figure 1 shows 12 sample images from the collected dataset.

### 2.2. Collection Procedure

Imaging with light wavelengths in the near-infrared region (NIR) aids in the detection of veins with high contrast. This is because the haemoglobin protein in the blood absorbs NIR light, whereas the skin and subcutaneous tissue above veins easily pass it through [15].

**Figure 2:**
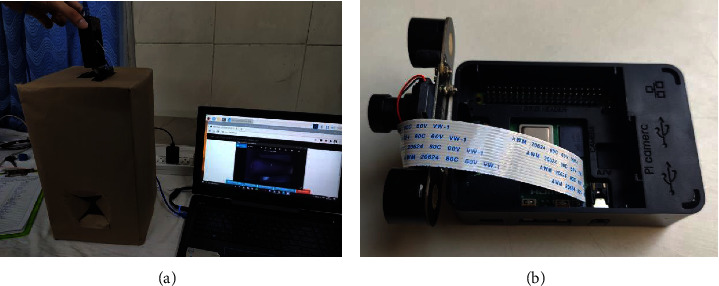
Data acquisition setup showing (a) Cardboard box with holes for arm and camera and (b) Camera setup mounted on raspberry Pi case.

To take advantage of this property, the imaging procedure was carried out by simulating ambient lighting conditions inside a closed cuboidal cardboard box measuring 210 mm × 210 mm × 400 mm. 2 circular holes with a diameter of 110 mm were cut out at opposite ends at a depth of 220 mm from the top. The cut-out areas were then covered with dark coloured paper, thus ensuring minimal external light to enter the box. In order to capture images, a setup consisting of two 850 nm and 1 W power near-infrared Light Emitting Diodes (LEDs) mounted on either side of a 5-megapixel OV5647 (OmniVision) camera [16] was used. This setup was then mounted on the plastic encasing of the Raspberry Pi, a low cost microcomputer which was used to power and operate the camera, by means of double tape. The entire apparatus (Figure 2(b)) was held by hand allowing the LEDs and camera to peek into the box through a small rectangular window of size 8 mm × 6 mm cut on the top, as shown in Figure 2(a).

The participants were asked to tightly clench their fist and pass their arm through the circular cavities till the elbow joint, allowing the anterior forearm to face the boxes' ceiling, allowing the camera to capture an image from above. They were then asked to further insert their arm into the column, in order to capture the veins of the antecubital fossa region. This process was repeated for the other arm, hence a total of 4 images were captured per participant. No tourniquet or any other sort of pressure was applied on the upper arm while capturing the images.

## 3. Image Processing

Computer Vision image processing techniques may be utilised in a variety of medical imaging systems for augmentation, analysis, and noise reduction. Furthermore, the approaches listed below have been shown to be effective in extracting information from pictures:

1. Noise removal
2. Edge/boundary enhancement
3. Contrast/transformations

The collected images were subjected to both local and global image processes, which are explained in Section 3.1.

### 3.1. Image Thresholding

Image thresholding as a technique is used in scenarios where a distinction between the brighter and comparatively darker pixels in an image is required. Based on a given threshold value, the pixels of an image are either set high or low. It is useful in localizing image segments in linearly separable images, but can also be used to make a distinction between the lighter and darker regions of an image. (1) explains the mathematical implication of the same.

(1) $$\begin{array}{c} g\left(x,y\right)=\left\{1,f\left(x,y\right)>T0,f\left(x,y\right)\leq T\right.\text{,} \end{array}$$

where *T* is the threshold value, and *x* is the current pixel value ranging between 0 and 255 in an 8 bit grayscale image.

### 3.2. Adaptive Histogram Equalization (AHE)

**Figure 3:**
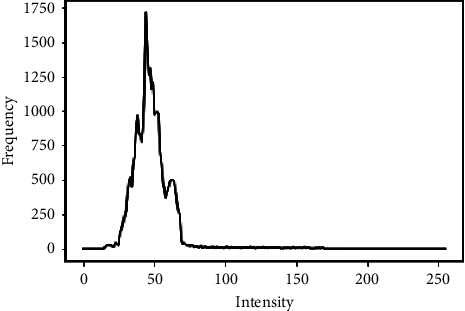
Sample image histogram.

**Figure 4:**
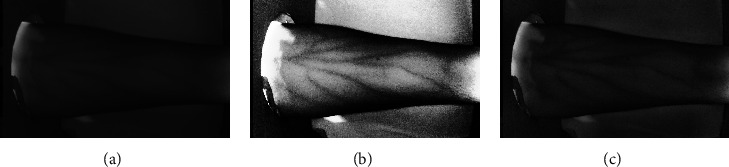
Resulting images after applying (b) AHE and (c) CLAHE techniques on the original image (a).

This method is frequently used to enhance image contrast amplification. A pixel is modified using the histogram of nearby pixels in Adaptive Histogram Equalization. This technique is useful for increasing the global contrast of an image when its pixel values lie within a close range. Hence, it is a good choice for boosting local contrast and sharpening edge definition in each image region.  Figure 3 illustrates the histogram of a black and white image denoting the count of pixel values as the ordinate and the relative brightness (0–255) on the abscissa.  Figure 4(b) shows the resulting image after applying AHE filter on the original image (Figure 4(a)).

### 3.3. Contrast Limited Adaptive Histogram Equalization (CLAHE)

The CLAHE method is comparable to picture transformations based on Adaptive Histogram Equalization. Because of the comparable nature of neighbour pixels, Adaptive Histogram Equalization frequently contributes to noise in the constant contrast based regions of the image. To further amplify the noise reduction, the slope of the transformation function is used to transform a pixel value.

Contrast reduction of an image through CLAHE follows a sequential flow of control involving the following processes:

1. Tiling
2. Applying Equalization through histograms
3. Bi-linear Interpolation

**Figure 5:**
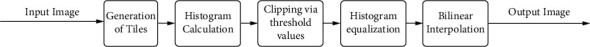
Sequence of steps followed for applying contrast limited adaptive histogram equalization.

The image is initially split into tiles, which are discrete fixed-area sections. To perform Histogram Equalization, a clip limit is determined to inhibit the noise amplification. Following this, for each tile a histogram is computed and values greater than the clip limit are readjusted into the neighbour tiles. For contrast adjustment, the individual images are nested together with the help of Bilinear Interpolation. This procedure ensures that the regions with darker sections are not over amplified and can cause erroneous predictions, especially when working with biomedical data. The overall sequential flow of control is illustrated in Figure 5. The effect of applying CLAHE on an image (Figure 4(a)) can be seen in Figure 4(c).

## 4. Experiments

To determine the usability of the presented dataset, image segmentation was carried out using an architecture based on the state-of-the-art deep learning technique, U-Net [13] on 50% of the dataset. The images were annotated so to have 2 classes for each pixel, either “vein” or “background.”

This section discusses the architecture of the U-Net model, the training procedure followed and the evaluation metrics used to produce the results presented in Section 5.

### 4.1. Proposed U-Net Model

#### 4.1.1. Model Architecture

**Figure 6:**
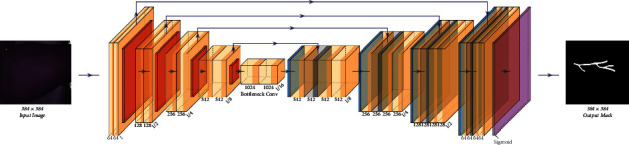
Architecture of the U-Net model.

The encoder has been used as a contraction path that decreases the size of the image continuously from 384 × 384 × 3 to 24 × 24 × 1024 whilst the depth gradually increases. It entails applying 3 × 3 convolution operations (unpadded) many times, each followed by a rectified linear unit (ReLU) layer and a 2 × 2 max pooling operation with stride 2 for down sampling which helps in extracting the information from images. For localizing the gain in information, an expansion path based decoder has been utilised which increases the image size from 24 × 24 × 1024 to 384 × 384 × 1 and decreases the depth. An up sampling of the feature map is done by a 2 × 2 convolution (“up-convolution”) at each step along the expanded route. This is followed by two 3 × 3 convolutions and a rectified linear unit (ReLU) operation across the depth. The contraction and expansion paths have been visualised in Figure 6.

#### 4.1.2. Model Training

For training the model, an 8 : 1 : 1 split was used for training, validation and test set, respectively, using an image size of 384 × 384 and a batch size of 16. The model was trained for 100 epochs keeping the learning rate constant at 0.0001.  Table 1 summarises the hyper-parameters and architecture of the model. Basic image transformations such as shearing, flipping, and custom cropping were used to extract more input samples from the given set of images. The following augmentation procedures were applied to compare the performance of the filtering methods discussed in Section 3:

1. No augmentations
2. Only AHE
3. Only CLAHE
4. Both AHE & CLAHE

**Figure 7:**
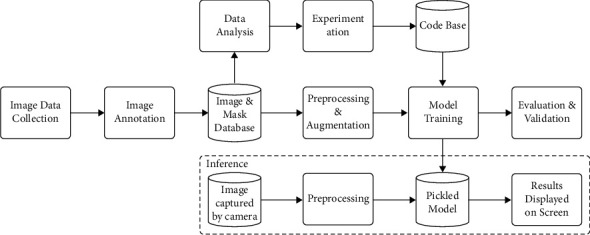
A schematic diagram illustrating the entire workflow.

The entire process from data collection to model inference is shown in Figure 7 in the form of a flowchart.

### 4.2. Evaluation Metrics

#### 4.2.1. Peak Signal-to-Noise Ratio (PSNR)

The PSNR is a signal processing measurement that compares a given received or processed signal to its original source signal. It has been shown that it is a quality evaluation method for image segmentation [17]. The PSNR between the ground truth (*I*) and predicted mask (*K*) is described by equations (2) & (3) as follows:

(2) $$\begin{array}{c} PSNR=20\frac{{\mathrm{MAX}}^{2}}{\sqrt{MSE}}\text{,} \end{array}$$

(3) $$\begin{array}{c} MSE=\frac{1}{mn}\overset{m-1}{\underset{i=0}{\sum }}\overset{n-1}{\underset{j=0}{\sum }}{\left[I\left(i,j\right)-K\left(i,j\right)\right]}^{2}\text{,} \end{array}$$

where MAX is the maximum possible value of the signal, being 255 for an 8 bit grayscale image.

#### 4.2.2. Intersection over Union (IoU)

The Intersection over Union (IoU) metric traditionally referred to as the Jaccard Score, is a method to calculate the percent overlap between 2 images. The IoU score between the ground truth (*X*) and predicted mask (*Y*), is defined by equation (4) as follows:

(4) $$\begin{array}{c} IOU\left(X,Y\right)=\frac{X\cap Y}{X\cup Y}\text{.} \end{array}$$

#### 4.2.3. Dice Coefficient

Dice coefficient is a measure of similarity between two images. It is a standardized metric for evaluating segmentation models. The Dice score is given by (5) as follows:

(5) $$\begin{array}{c} Dice\left(X,Y\right)=\frac{X\cap Y}{\left|X\right|+\left|Y\right|}\text{.} \end{array}$$

## 5. Results

To evaluate the performance of the 4 models trained with/without AHE and CLAHE augmentations, an unseen set of 93 images not used in the training or validation was used as a test set. It can be inferred from Table 2 that the inclusion of augmented images in the training procedure shows an improvement in the PSNR, IoU, and Dice scores. Furthermore, keeping other hyper-parameters constant, the training procedure was repeated to experiment with different values of epochs, learning rate, and different activation functions.  Table 3 summarises the performance of the model for different values of these hyper-parameters.

**Figure 8:**
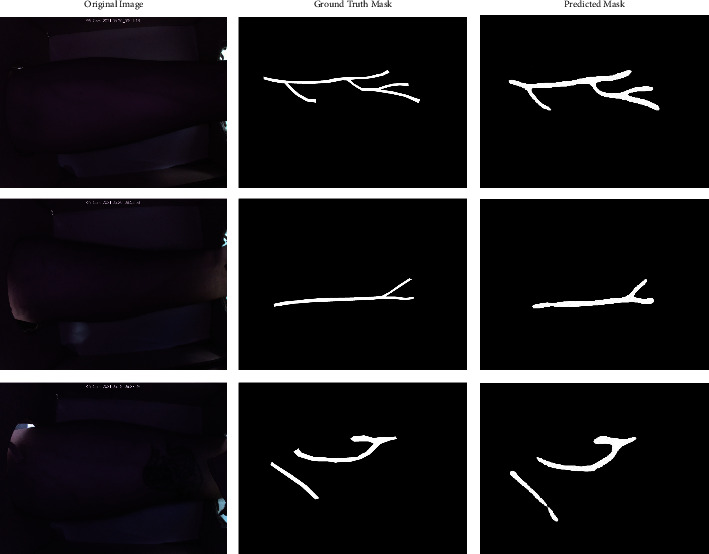
Input image, annotated ground truth, and predicted output from U-Net model for 3 sample images.

**Figure 9:**
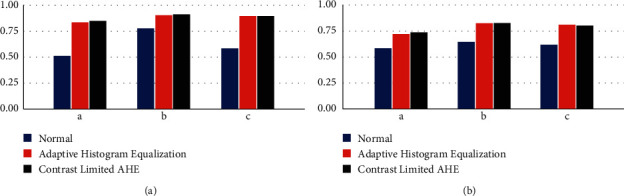
Comparing the effect of test-time augmentations on 3 images based on their (a) Dice coefficient and (b) IoU.

**Figure 10:**
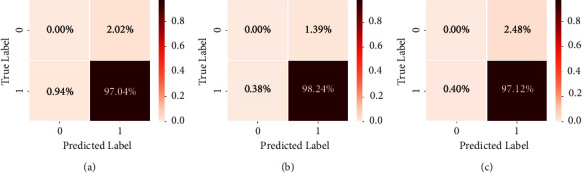
Image wise performance analysis using a confusion matrix.

Figure 8 shows a set of 3 raw images from the dataset, their annotated true mask, and the predicted output mask from the model. It can be observed that the predictions obtained have a high resemblance to the vascular patterns visible in the input image. The 3 raw images were subjected to CLAHE and AHE filtering and passed to the U-Net model for processing. It can be seen that the application of test-time augmentations shows a drastic improvement in the Dice (Figure 9(a)) and IoU (Figure 9(b)) scores. Confusion matrices corresponding to the 3 images have been drawn below in Figure 10 to provide better insights. It could be observed that the predictability of a pixel as a vein wherein a vein exists, is high. Correspondingly, the probability of a nonvenous region being predicted as a vein is as low as ∼0.002.

While comparing the results of the models trained with AHE and CLAHE, it can be noticed that CLAHE performs evidently better than AHE on the same set of images. For images with AHE, the over amplification of vein patterns the corresponding vein predictions come out to be relatively thicker than their corresponding masks which limits the values for IoU and Dice Coefficient. CLAHE on the other hand performs better because it inhibits the excessive enhancement of relatively darker pixel values.

## 6. Conclusions and Future Work

Even though the forearm region is one of the most common sites for intravenous (IV) procedures, the availability of forearm vein image datasets is scarce. Hence, a high quality primary dataset of the forearm has been presented in this paper. The methods used for data collection have been discussed emphasizing how NIR imaging combined with certain image processing techniques make it possible to visualise veins with clarity and distinction. Further, image segmentation was carried out to identify the location of veins in the image, and the discussed filtering methods were compared against each other.

### 6.1. Future Work

The comparison done in Table 3 has been done using the conventional classification metrics and loss functions. Training parameters and evaluation could further be extended using more recent metrics and loss functions such as the Lovasz-Softmax Loss [18] for optimization.

The performance of the algorithm presented could further be improved upon in the future with better quality annotations and larger architectures to facilitate the understudy. The presented dataset can be used for vein detection and segmentation. In addition, this technique could be used as a base architecture to extend the usability of an algorithm that can be used for standalone intravenous imaging.

The detected veins can be prioritized on the basis of intrinsic physical parameters such as thickness, infrared temperature measurement, or depth to detect a venipuncture point which can be used to assist in IV procedures such as cannulation and blood extraction. Moreover, the said detected venipuncture site can contribute to the research and development of devices which aim to automate IV procedures with the aid of computer vision, in which the closed contraption of the device would provide the functionality similar to the cardboard box used in the imaging technique proposed in this study. Furthermore, the presented dataset can be used to train segmentation models which would expedite the development of the said devices.

## Figures and Tables

**Table 1:** Default parameters of the mode.

Parameters	Value
Image size	384^*∗*^384
Learning rate	1*e*–4
Epochs	100
Image colour mode	RGB
Mask colour mode	Greyscale
No. of convolutional blocks	4
No. of de-convolutional blocks	4

**Table 2:** Comparison of different augmentation methods on the basis of their PSNR, IoU, and dice scores.

	PSNR				IoU				Dice			
Training with:	Mean	Std	Min	Max	Mean	Std	Min	Max	Mean	Std	Min	Max
No augmentations	0.435	0.146	0.068	0.678	0.682	0.01	0.552	0.961	0.397	0.11	0.067	0.517
AHE	0.582	0.165	0.045	0.827	0.788	0.007	0.659	0.973	0.48	0.141	0.045	0.708
CLAHE	0.624	0.165	0.097	0.886	0.79	0.005	0.718	0.98	0.545	0.146	0.097	0.799
Both AHE & CLAHE	**0.751**	0.155	0.117	0.93	**0.893**	0.004	0.821	0.996	**0.685**	0.149	0.117	0.871

**Table 3:** Performance of the U-Net model with varying hyper-parameters.

	Epochs			Learning rate			Activation function		
Metric	50	100	200	0.0001	0.0005	0.001	Tanh	Sigmoid	ReLU
Dice coefficient	0.52	0.685	0.678	0.685	0.572	0.542	0.532	0.468	0.685
IoU	0.818	0.893	0.895	0.893	0.853	0.847	0.826	0.784	0.893
PSNR ratio	0.619	0.751	0.741	0.751	0.596	0.583	0.581	0.505	0.751

## Data Availability

The experimental data used to support the findings of this study are available from the corresponding author upon request.
